# Reducing Cathodic Drift during Isoelectric Focusing Using Microscale Immobilized pH Gradient Gels

**DOI:** 10.1021/acs.analchem.4c00788

**Published:** 2024-05-08

**Authors:** Gabriela Lomeli, Amy E. Herr

**Affiliations:** † The UC Berkeley−UCSF Graduate Program in Bioengineering, University of California, Berkeley, California 94720, United States; ‡ Department of Bioengineering, University of California, Berkeley, California 94720, United States; § Chan Zuckerberg Biohub, San Francisco, California 94158, United States

## Abstract

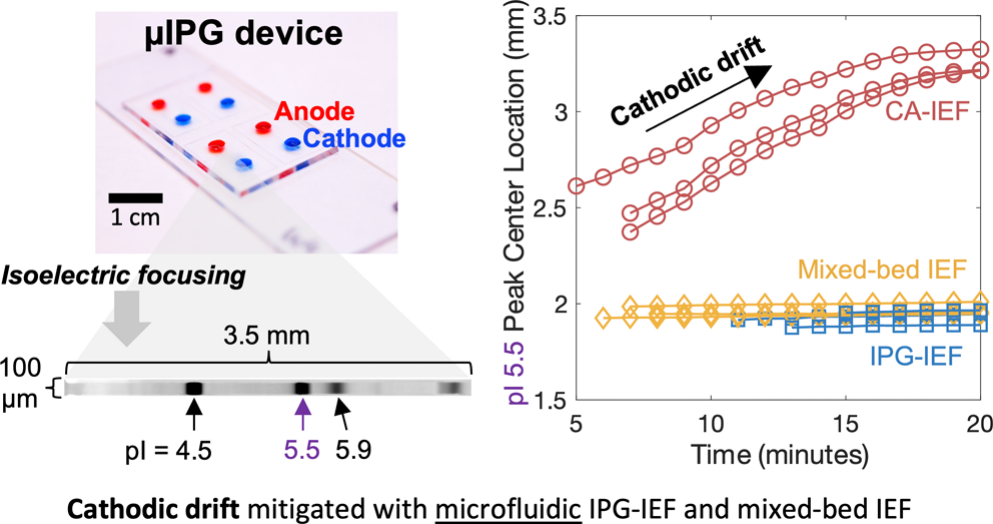

Microfluidic analytical tools play an important role in miniaturizing targeted proteomic assays for improved detection sensitivity, throughput, and automation. Microfluidic isoelectric focusing (IEF) can resolve proteoforms in lysate from low-to-single cell numbers. However, IEF assays often use carrier ampholytes (CAs) to establish a pH gradient for protein separation, presenting limitations like pH instability in the form of cathodic drift (migration of focused proteins toward the cathode). Immobilized pH gradient (IPG) gels reduce cathodic drift by covalently immobilizing the pH buffering components to a matrix. To our knowledge, efforts to implement IPG gels at the microscale have been limited to glass microdevices. To adapt IEF using IPGs to widely used microfluidic device materials, we introduce a polydimethylsiloxane (PDMS)-based microfluidic device and compare the microscale pH gradient stability of IEF established with IPGs, CAs, and a hybrid formulation of IPG gels and CAs (mixed-bed IEF). The PDMS-based IPG microfluidic device (μIPG) resolved analytes differing by 0.1 isoelectric point within a 3.5 mm separation lane over a 20 min focusing duration. During the 20 min duration, we observed markedly different cathodic drift velocities among the three formulations: 60.1 μm/min in CA-IEF, 2.5 μm/min in IPG-IEF (∼24-fold reduction versus CA-IEF), and 1.4 μm/min in mixed-bed IEF (∼43-fold reduction versus CA-IEF). Lastly, mixed-bed IEF in a PDMS device resolved green fluorescent protein (GFP) proteoforms from GFP-expressing human breast cancer cell lysate, thus establishing stability in lysate from complex biospecimens. μIPG is a promising and stable technique for studying proteoforms from small volumes.

Isoelectric focusing (IEF) is an equilibrium electrophoretic protein separation designed to resolve proteoforms, including proteins having an array of post-translational modifications (PTMs).^1,2^ During IEF, sample proteins electromigrate within a pH gradient and focus at the isoelectric point (pI) of each respective species. The pI is the pH at which a molecule has zero net charge, resulting in no electrophoretic mobility.^3^ PTMs, such as phosphorylation and proteolytic cleavage, can alter the pI of a protein, making IEF an appropriate separation technique.^4^ For instance, phosphorylation involves the addition of a negatively charged phosphate group to an amino acid, consequently reducing the pI of the protein.^4^ The degree of change in pI is contingent upon factors like the number of PTM events, the specific amino acids involved, and the overall protein composition.^4^

Since the inception of IEF, miniaturization has been pursued as a means to achieve high-throughput analyses with reduced starting sample amounts, including for single-cell IEF analyses.^2,5^ However, as IEF progresses toward the microscale, the increasing challenge of maintaining a stable pH gradient has limited the utility of this targeted proteomics tool.^6−8^ IEF can be achieved through various methods, such as using pH gradients generated via water electrolysis^9^ or thermal gradients.^10^ Even with the introduction of novel methods for creating pH gradients, chemistry-based IEF technologies remain commonly used.^11^ Here, we consider the following: carrier ampholyte IEF (CA-IEF),^12^ immobilized pH gradient IEF (IPG-IEF),^13^ and mixed-bed IEF (a hybrid of CA-IEF and IPG-IEF).^14−16^

In CA-IEF, bracketed by anolyte (acidic) and catholyte (basic) regions and subjected to an applied electric field, a mixture of CAs arrange into an increasing pH gradient within an anticonvective matrix, such as polyacrylamide (PA) gel.^17^ CAs are a mixture of small molecules with both positive and negative charge groups (amphoteric). CAs serve as conductors of electrical current and act to buffer the pH.^17^ CAs are generated with a chaotic synthesis method to generate hundreds of molecules capable of assembling into a monotonically increasing pH gradient.^12^ Microfluidic IEF typically employs CA-IEF.^2,6,7,18^ Unfortunately, CA-IEF suffers from pH gradient instability in the form of cathodic drift.^19^ Cathodic drift is the observed movement of sample and other separation components toward the cathode, resulting in an overall shift in the pH gradient.^20^ The underlying mechanism of cathodic drift is attributed to several factors, including CA electromigration, electro-osmotic flow (EOF) due to the slight negative charge of the PA gel, and electrolyte diffusion.^21^ Cathodic drift leads to challenges during IEF, including loss of separation resolution^22^ and loss of proteins with high pH as the proteins run off the separation lane.^7^ A prior microfluidic IEF study found >3-fold improvement in separation resolution when cathodic drift was reduced >20-fold.^7^ While work has been done to reduce cathodic drift in centimeter-scale slab IEF to a manageable cathodic drift velocity (∼100 μm/min),^23^ the cathodic drift velocity in microscale devices (∼10–600 μm/min)^18,21,24,25^ is more pronounced relative to the characteristic length (micrometers) and time scales (minutes) for microscale IEF separation.^6^

IPG-IEF was developed to improve pH gradient stability over CA-IEF.^19^ In IPG-IEF, a pH gradient is established prior to sample application by copolymerizing a class of buffering molecules known as Immobilines into PA gel. By creating a concentration gradient of Immobilines at the time of polymerization, an immobilized pH gradient (IPG) gel is established. Unlike CAs, Immobilines are not amphoteric molecules, but are instead either weak acid or weak base acryloyl monomers that can be polymerized into the PA gel to participate in protolytic equilibria to perform a buffering function.^26^ Cathodic drift is reduced or even eliminated in IPG-IEF because the Immobilines are polymerized into the PA gel and therefore insolubilized, thus the resultant pH gradient remains fixed in space and does not drift under the action of an applied electric field.^26,27^ A microfluidic IPG (μIPG) device was previously developed in glass microchannels.^28^ While glass microfluidic devices are a cornerstone of early microfluidic advances and have achieved commercial success,^29^ a rising trend among microfluidic researchers is the shift toward rapid prototyping using polymer-based devices, particularly PDMS devices, as invaluable research tools.^30,31^

While effective at mitigating cathodic drift, IPG-IEF requires longer focusing times and larger sample amounts compared to CA-IEF due to the lower conductivity of the IPG-IEF separation environment, resulting in slowed protein migration.^15,16,32^ Another IEF technology, mixed-bed IEF, was introduced to combine the advantages of both IPG- and CA-IEF.^19^ In mixed-bed IEF, a stationary, Immobiline-driven pH gradient coexists with a soluble, CA-driven pH gradient, which increases the conductivity of the separation environment versus IPG-IEF alone.^15^ Mixed-bed IEF has advantages over IPG-IEF with regards to focusing speed and sensitivity^15,16^ while retaining the pH gradient stability and cathodic drift suppression characteristic of IPG-IEF.^19^ Disadvantages of mixed-bed IEF versus IPG-IEF stem from the use of CAs in mixed-bed IEF. CAs increase both the cost and complexity of the mixed-bed IEF assay relative to IPG-IEF while also introducing the potential for adverse protein-CA interactions.^8^ In mixed-bed IEF, suppression of cathodic drift is hypothesized to arise from the maintenance of isoelectric pH and the position of the soluble CAs by the buffering mechanism of Immobiline molecules covalently bound to the PA gel.^15,33^ A previous study demonstrated that cathodic drift stemming from EOF could be reduced by neutralizing the negative charges of PA gel.^23^ Neutralization was achieved by covalently incorporating a tertiary amine, 3-(dimethylamino)propylmethacrylamide (DMAPMA), within the PA gel in equimolar proportions to the PA gel’s negative charges.^23^ Immobilines in mixed-bed IEF are hypothesized to exert a similar effect as DMAPMA in buffering the negative charges of PA, thereby mitigating EOF-induced cathodic drift.^33^ Importantly, while several groups have accomplished microfluidic CA-IEF^2,6,7,18^ and IPG-IEF,^28^ prior efforts to implement microscale mixed-bed IEF have been unsuccessful for reasons that were not elaborated,^28^ a question explored in the present study.

In this work, we consider polydimethylsiloxane (PDMS) microdevices, hereafter referred to as μIPG devices, as platforms for IPG-IEF and mixed-bed IEF. We explore PDMS as a separation device substrate owing to PDMS’s cost-effective and rapid prototyping potential and biocompatibility.^34^ First, we devise a protocol to fabricate IPG gels in PDMS microchannels, borrowing benzophenone-assisted PA gel polymerization from our earlier reported work.^31^ We next investigate the mitigation of cathodic drift in the μIPG devices when operated with CA-IEF, IPG-IEF, or mixed-bed IEF modes. Finally, we perform mixed-bed IEF in the μIPG device for analysis of green fluorescent protein (GFP) proteoforms from crude mammalian cell lysate.

## Experimental Section

### μIPG Device Design

The μIPG device was fabricated by standard soft lithography methods. The PDMS substrate was patterned with four microchannels and assembled onto a glass slide. Each microchannel is 3.5 mm long, 100 μm wide, and 50 μm tall. We apply Kapton tape on the glass slide opposite of the PDMS substrate to serve as a photomask and prevent photopolymerization in those regions. A detailed protocol is described in the Supporting Information.

### Polyacrylamide Gel Fabrication

To polymerize PA gel in the PDMS channels, we adapted a technique from previous work that ensures gel polymerization in a microchannel environment that may be O_2_ rich.^31^ Briefly, the microchannels are treated with benzophenone before any gel precursor is applied. Benzophenone serves as an oxygen scavenger when exposed to UV.^35^ Next, a 6%T PA gel precursor is applied at one of the microchannel inlets. Table S1 summarizes the PA gel precursor recipes. The scaffold PA gel precursor is then photopolymerized with UV light. The UV activates both the benzophenone (oxygen scavenger) and the photoinitiator in the PA gel precursor (radical polymerization initiator).

The two inlets on both ends of the microchannel are then emptied and filled with acidic (pH = 3.8) and basic (pH = 7.0) Immobiline–PA gel precursor solutions, respectively. Acidic Immobilines used were acrylamido buffers p*K*_a_ 3.6 and 4.6. Basic Immobilines used were acrylamido buffers, p*K*_a_ 6.2, 7.0, and 9.3. The acidic and basic IPG precursor recipes were adapted from previous work,^28^ and either a 6%T or 12%T IPG gel formulation was used (see Table S1 for the IPG precursor recipes). The precursors were allowed to diffuse into the microchannels for 7 h to establish a linear concentration gradient along the length of the microchannel (see the Establishing a Linear pH Gradient subsection for details on how a linear gradient at 7 h was verified). The μIPG device was placed in a humidity chamber during the 7 h diffusion step to prevent evaporation. During this diffusion step, the polymerized 6%T PA gel serves as a scaffold for the IPG gel, maintaining quiescent conditions during gel precursor diffusion.^25^ The IPG precursor was then photopolymerized with UV light. The devices were incubated with (for CA-IEF or mixed-bed IEF) or without (for IPG-IEF) 1% ZOOM carrier ampholytes pH 4–7 dissolved in DI water or sample loading buffer overnight (as summarized in Table S2) and could be stored at 4 °C for at least 9 days. Detailed PA gel precursor reagents, photopolymerization conditions, and the composition of the sample loading buffer are described in the Supporting Information. Modifications to the PA gel fabrication protocol for pH gradient characterization and polymerization experiments are also detailed in the Supporting Information.

### Cell Culture and Cell Lysate Preparation

An MCF7 human breast cancer cell line genetically modified to stably express enhanced green fluorescent protein (GFP) was obtained from the American Type Culture Collection and maintained using standard cell culture practices. Cell lysate preparation was performed as previously described^18^ with some modifications. A detailed procedure is described in the Supporting Information.

### Isoelectric Focusing Experiments

Fluorescent pI markers (pIs 4.5, 5.5, 5.9, 6.6, and 6.7) were used at various concentrations to provide similar intensities. For cell lysate experiments, 1 μL of MCF7-GFP cell lysate (∼20 000 cells/μL) was applied to both the anode and cathode inlets (∼40 000 cells total), as well as pI markers in sample loading buffer. Table S3 lists the anode inlet and cathode inlet sample components for all IEF experiments. An electric field was applied using the following electric field ramp over 20 min of IEF: 50 V/cm (0–4 min), 100 V/cm (4–9 min), 200 V/cm (9–14 min), and 300 V/cm (14–20 min). The detailed IEF procedure is described in the Supporting Information.

### Imaging and Image Analysis

Microscope information for the fluorescence imaging as well as the methods for image/micrograph analysis are described in the Supporting Information.

## Results and Discussion

### Design of μIPG for Isoelectric Focusing

**Figure 1:**
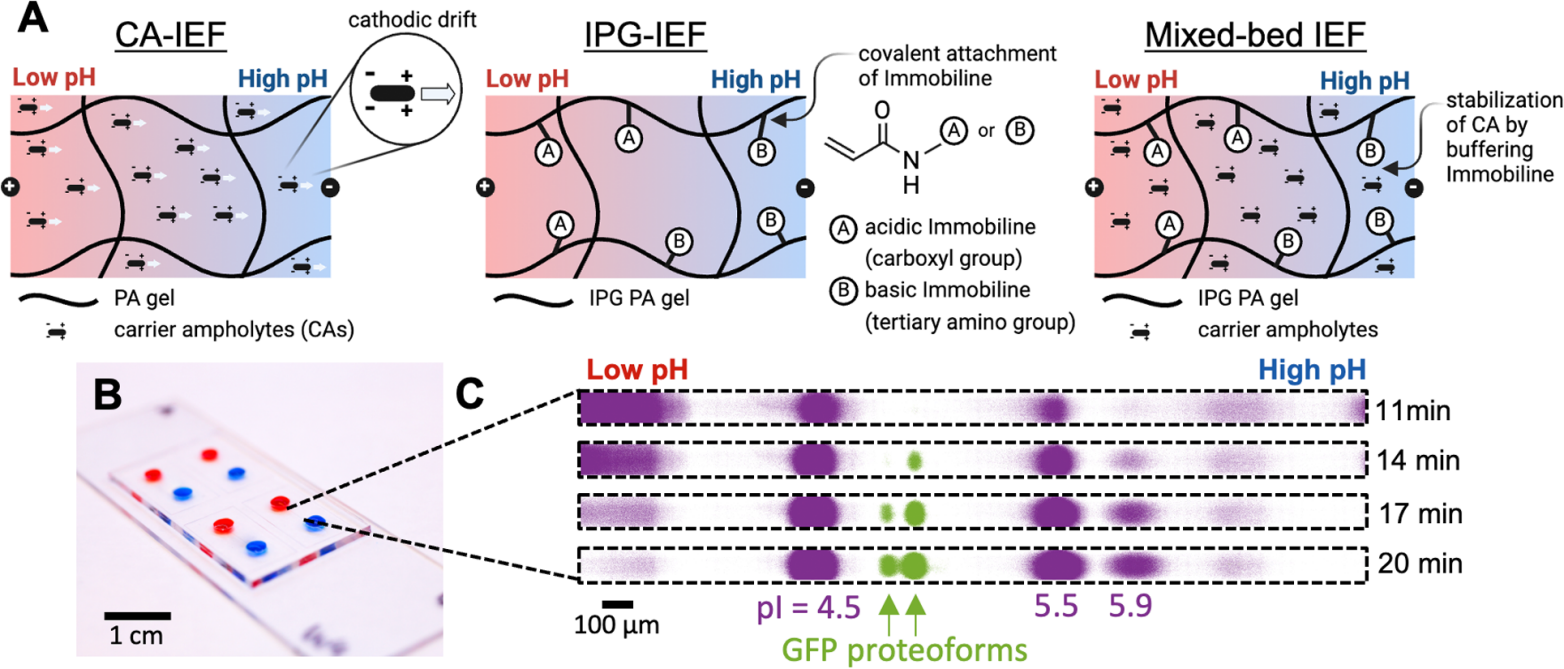
μIPG is designed to mitigate cathodic drift during IEF at the microscale for stable pI-based separation of proteoforms. (A) Schematic of pH buffering mechanisms for CA-IEF, IPG-IEF, and mixed-bed IEF. In CA-IEF, the pH is buffered by carrier ampholytes, which are subject to cathodic drift (indicated by light blue arrows). In IPG-IEF, Immobilines buffer the pH and cathodic drift is mitigated by covalent attachment of Immobilines to the PA gel. In mixed-bed IEF, the Immobilines stabilize the CA pH gradient to reduce cathodic drift. (B) μIPG device containing food-dye-filled microchannels. (C) Inverted fluorescence micrographs of mixed-bed IEF-separated pI markers (pIs 4.5, 5.5, and 5.9) and two GFP proteoforms at several time points demonstrating pH gradient stability, as the pI marker and protein peaks remain fixed in position over time. Micrographs have the same acquisition settings, brightness, and contrast. Representative of *n* = 3 separations. IPG gel is 6%T.

The IEF device can be operated using CA-IEF, IPG-IEF, or mixed-bed IEF (Figure 1A). The IEF device comprises four separation lanes within the footprint of a standard microscope slide (Figure 1B). During IEF, loaded analytes migrate along the pH gradient within the separation lane until each analyte reaches the location in the pH gradient where net zero charge arises in the molecule (Figure 1C).

**Figure 2:**
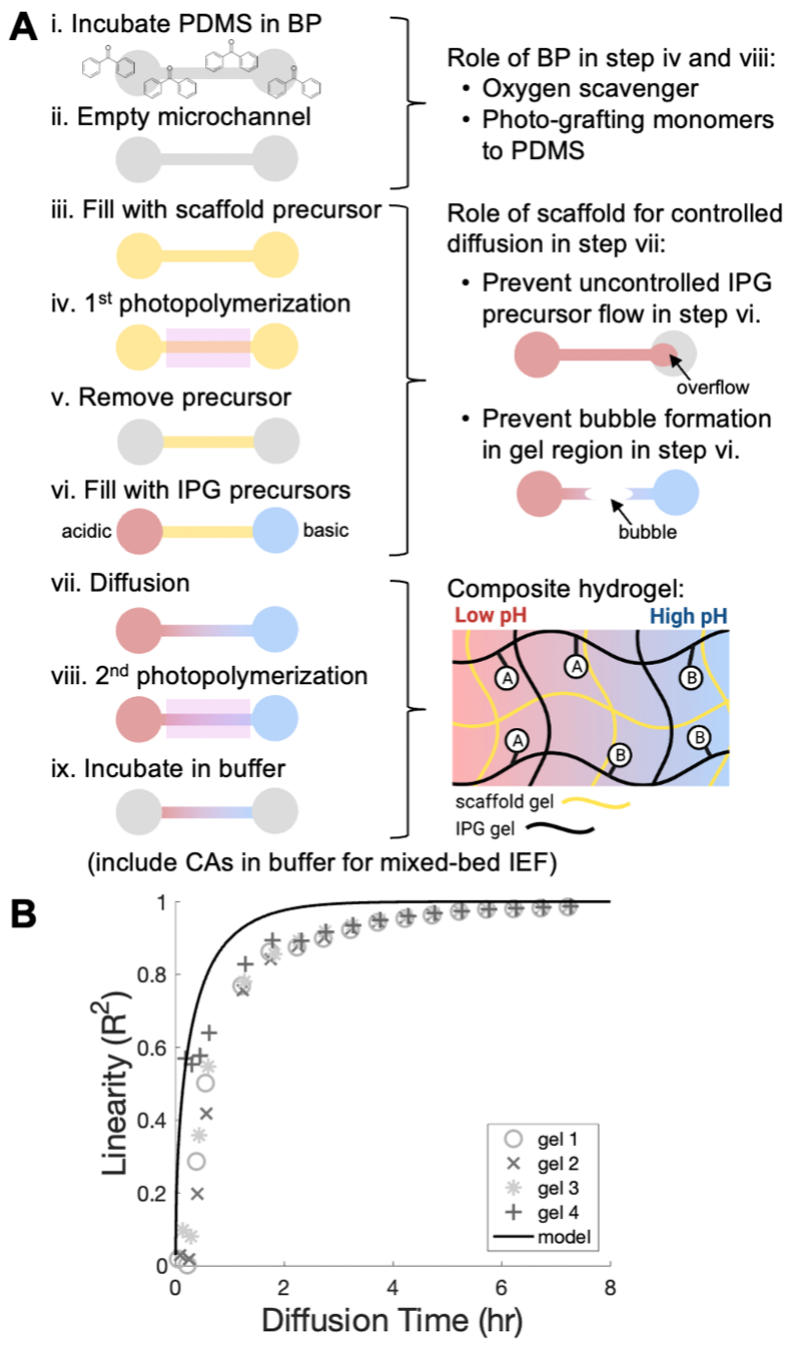
Design and fabrication of the μIPG device. (A) μIPG device fabrication protocol using a double-photopolymerization method. (B) Linearity of the gradient the during diffusion step is modeled and experimentally monitored (*n* = 4 gels) with Nile Blue acrylamide dye.

We first sought out to validate IPG-IEF in our IEF device before incorporating CAs for mixed-bed IEF. To fabricate a scaffold and IPG gel, Figure 2A reports a double-photopolymerization process. Steps i–v establish a scaffold gel within the PDMS microchannel, while steps vi–ix overlay the IPG onto the PA scaffold through diffusion, creating a composite hydrogel/interpenetrating network. The scaffold gel prevents fluid flow and enables the establishment of a linear Immobiline gradient in steps vii. A similar strategy was previously employed to create an on-chip pore-size gradient in PA gel.^25^ UV irradiation is used in both photopolymerization steps for two reasons. First, the UV activates the benzophenone previously absorbed into the PDMS to quench oxygen and graft the PA gel to the PDMS.^35^ Second, the UV activates the photoinitiator, VA-086, in the gel precursors to initiate radical PA polymerization. We confirmed that all precursors polymerized in the PDMS channel and that both UV irradiation and benzophenone were necessary for polymerization (Figure S1). Therefore, we overcome the challenge of performing radical chemistry in PDMS by utilizing an oxygen scavenger.^35^ Finally, the choice of buffer in step ix dictates whether the device is operated as IPG-IEF (no CAs in the buffer) or mixed-bed IEF (CAs included in the buffer).

### Establishing a Linear pH Gradient

We sought to establish a linear Immobiline concentration gradient within the μIPG microchannel, which in turn creates a linear pH gradient. IPG pH gradients are highly tunable, including the option to form a linear or nonlinear gradient.^19^ A nonlinear pH gradient may be desired to create regions of narrow pH to analyze complex protein mixtures.^36,37^ To demonstrate the feasibility of IPG-IEF in a PDMS device, the present work focuses on linear pH gradients.

To determine the diffusion time necessary to establish a linear gradient of IPG gel precursor molecules within a microchannel prefilled with 6%T PA scaffold gel, we modeled the diffusion behavior of a proxy molecule, Nile Blue acrylamide, that is detectable using real-time fluorescence imaging (unlike Immobiline molecules). Since Nile Blue acrylamide has a similar, yet larger, molecular mass than all the individual molecules of the IPG gel precursors, we can therefore assume Nile Blue acrylamide will have a similar or slower diffusion rate than the IPG gel precursor molecules we want to model. The diffusion coefficient for Nile Blue acrylamide is calculated to be 0.0162 mm^2^/min (Note S1).

Next, the concentration profile of Nile Blue acrylamide in the microchannel was modeled with the 1D diffusion equation (Note S1). We compared the numerical modeling results to experimental results and observed that diffusion in the microchannel was slower than predicted (*R*^2^ > 0.95 achieved after ∼250 min instead of expected ∼120 min) (Figure 2B). We hypothesize that the slower effective Nile Blue acrylamide diffusion could be due to absorption of Nile Blue acrylamide by the PDMS,^38^ which we observed in our devices (data not shown). Based on our experimental results, the Nile Blue acrylamide concentration profile is linear after 7 h (Figure 2B). We therefore chose a diffusion time of 7 h as a conservative estimate of the time needed to establish a linear Immobiline gradient, which allowed device fabrication to be completed in 1 day.

**Figure 3:**
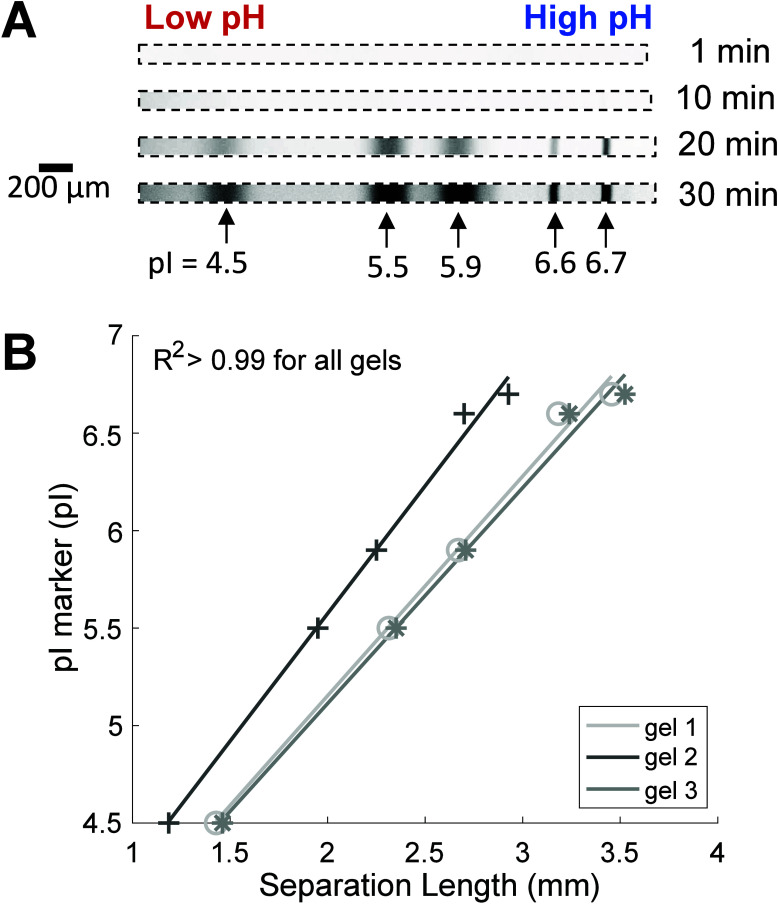
IEF of pI markers in a μIPG device confirms a linear pH gradient. (A) Inverted fluorescence micrographs of IEF-separated pI markers at several time points demonstrate separation evolution and stability. Micrographs have the same acquisition settings, brightness, and contrast. Representative of *n* = 3 separations. (B) Plot showing the 5 pI markers versus position to determine the linearity of the pH gradient after 20 min of IEF from pH 4.5–6.7 for *n* = 3 gels/separations. IPG gel is 6%T.

After fabricating μIPG devices with Immobiline reagents, we next sought to investigate the linearity of the pH gradient via IEF of a fluorescent pI marker ladder. Our μIPG device was designed to separate analytes having pIs in the pH range of 3.8–7.0. Figure 3A demonstrates separation of five pI markers (ranging from 4.5 to 6.7). The pH gradient in the μIPG device is linear from 4.5 to 6.7 (Figure 3B). We observe device-to-device variation in the position of the pH gradient within the microchannel, as demonstrated by the leftward shift of gel 2’s pH gradient in comparison to the other two technical replicates (Figure 3B). To account for device-to-device variation leading to shifts in the pH gradient, we include fluorescent pI markers in all IEF runs to assess run success and as internal standards of pI. The use of pI markers as internal standards of pI in order to determine the pI of unknown species is routine in IEF.^39^ We hypothesize that this variation was caused by the visual alignment of the Kapton tape photomask to the microchannels during device fabrication. Reduction in device-to-device variability could be achieved using a mask aligner.

Notably, the μIPG device resolves pI markers 6.6 and 6.7, which differ by 0.1 pH unit (Figure 3). For context, a single phosphorylation event can cause a pI change of 0.3–0.4 pH units.^40^ We anticipate that even smaller pI differences can be resolved in the μIPG device for proteins (∼100-fold larger molecular mass than pI markers), since smaller molecular mass samples tend to experience more peak broadening and therefore a loss in separation resolution.^41^ The peak width is a function of the sample’s inherent diffusivity (*D*) and mobility slope (d*u*/d(pH)), as well as the electric field strength (*E*) and steepness of the pH gradient (d(pH)/d*x*).^17^ Accordingly, we note that the two most basic pI markers, pI markers 6.6 and 6.7, demonstrate narrower peak widths than the other pI markers (Figure 3), which could stem from any of the aforementioned variables and facilitates the resolution of the 0.1 pH unit difference.

In IEF, the minimum resolvable pI difference (Δ(pI)_min_) is a metric of separation resolution and is linearly proportional to d(pH)/d*x*.^17^ Since d(pH)/d*x* in IPGs is tunable, increased separation resolution (smaller Δ(pI)_min_) may be achieved by choosing a narrower pH range than the 3.8–7.0 pH range employed here. Critically, the performance of our PDMS-based μIPG device (Δ(pI)_min_ ∼ 0.1) is comparable to that of the previous glass-based μIPG device (Δ(pI)_min_ ∼ 0.04),^28^ even though the separation length in the PDMS-based μIPG device is 1.7-fold shorter (3.5 mm versus 6 mm), making our PDMS-based μIPG device the μIPG device with the shortest separation length to our knowledge. In addition to reducing the footprint of each separation, which can have advantages for increasing throughput, we anticipate that additional miniaturization of the μIPG device will facilitate analysis of small sample amounts by reducing the available surface area for nonspecific absorption of sample to the microchannel walls and anticonvective matrix. Moreover, since the peak positions are stationary (Figure 3A), the separation lane of the μIPG device can remain short compared to CA-based microfluidic devices, where the microchannel should be sufficiently long to prevent samples from running off the separation lane due to cathodic drift.^7^

### pH Gradient Stability and Performance of the μIPG Device

In IEF, pH gradient stability refers to the ability of the pH gradient established in the separation environment to remain consistent during sample focusing. pH gradient stability is particularly crucial in microscale devices, where even minor drift velocities (ranging from tens to hundres of μm/min) can have adverse effects on separations over micrometer-scale distances.^6^ To compare pH gradient stability in CA-IEF, IPG-IEF, and mixed-bed IEF, we monitored pI marker peak positions over time in our μIPG device for all three conditions. To perform an accurate comparison between IPG-IEF and CA-IEF in our device, the only modifications we made to the CA-IEF condition compared to the IPG-IEF condition were (1) Immobiline reagents were excluded from the PA gel and (2) the device was incubated in 1% CAs in DI water overnight. The mixed-bed IEF condition included Immobiline reagents and was incubated in 1% CAs in DI water overnight. Anode and cathode sample components were the same for all IEF conditions (Table S3).

**Figure 4:**
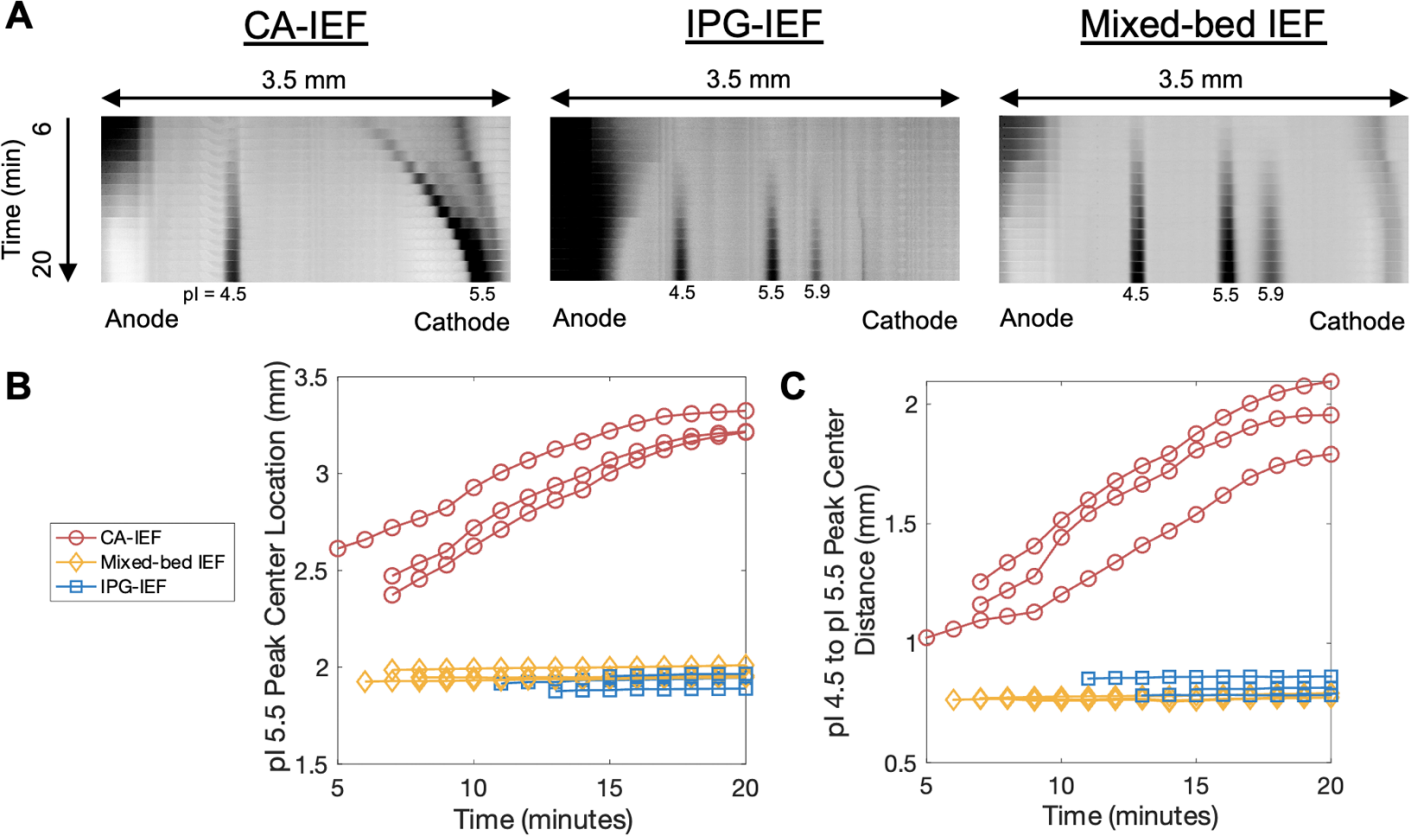
Cathodic drift in μIPG is reduced with IPG- or mixed-bed IEF versus CA-IEF. (A) Inverted fluorescence kymographs of IEF-separated pI markers demonstrating separation evolution. (B) Plot of pI 5.5 peak center location versus IEF focusing time shows that the peak center has a stable position for IPG-IEF and mixed-bed IEF (minimal cathodic drift), while the peak center drifts toward the cathode for the CA-IEF separations. (C) Plot of the distance between pI 4.5 and pI 5.5 peak centers versus IEF focusing time demonstrating pH gradient shape is more stable for IPG- and mixed-bed IEF versus CA-IEF. For (B) and (C), peak center data from *n* = 3 separations for each IEF condition are plotted for each time point with SNR > 3.

Figure 4A kymographs demonstrate a representative separation evolution for all three IEF conditions tested, and Figure 4B summarizes the peak drift behavior for pI marker 5.5 for all conditions. Intensity plots taken over 20 min of IEF for all three IEF conditions additionally support visualization of the peak profiles and peak drift behavior (Figure S2). From the data collected during 20 min of IEF (Figure 4), the cathodic drift velocities of pI marker 5.5 are 60.1 ± 7.7 μm/min for CA-IEF, 2.5 ± 2.5 μm/min for IPG-IEF (∼24-fold reduction versus CA-IEF), and 1.4 ± 0.4 μm/min for mixed-bed IEF (∼43-fold reduction versus CA-IEF). Notably, only the mixed-bed IEF condition, and not the IPG-IEF condition, was statistically different from the CA-IEF condition (*p* < 0.05, one-way ANOVA performed with Kruskal–Wallis test and posthoc Dunn’s multiple comparison test). Although the results did not reach statistical significance, considering the ∼24-fold differences in cathodic drift velocities between CA-IEF and IPG-IEF, as well as the visual mapping of the peak drift behavior (Figure 4), there is still evidence to suggest that the observed difference is meaningful from a practical perspective. Our μIPG device, operated with either IPG-IEF or mixed-bed IEF, demonstrates improved pH gradient stability compared to CA-IEF and previously published microscale CA-IEF devices (∼10–600 μm/min cathodic drift velocities).^18,21,24,25^

Interestingly, we also observe anodic drift for pI marker 4.5 in one of the CA-IEF conditions (Figure S2), which is another source of pH gradient instability.^20^ Additionally, CA-IEF in our device did not resolve pI marker 5.9. We hypothesize that cathodic drift caused pI marker 5.9 to “run off” the gel before pI marker 5.9 was concentrated enough to be detected. Moreover, assessing the pH gradient stability at both ends of the pH gradient in mixed-bed IEF requires a wider-range pI marker ladder, encompassing pIs less than 4.5 and greater than 5.9. Prior work reports cathodic drift in the basic (high pH) region of mixed-bed IEF.^15^ Corroborating these observations, we measured a diminishing intensity of an unidentified band in the basic pH region over a 20 min period of mixed-bed IEF (Figure 1C), potentially indicative of cathodic drift in the region of pH > 5.9. Alternative explanations for the diminishing band intensity include the dissociation of an innately fluorescent pI marker-pI marker complex (which constitutes the unidentified band) over time.

Next, we sought to assess whether the pH gradient compressed or expanded over the course of IEF for the three IEF configurations scrutinized. Since the CA-IEF condition lacked the third pI marker (pI marker 5.9) needed to measure the slope of the pH gradient (d(pH)/d*x*) as would assess pH gradient shape, we instead measured the distance between pI markers 4.5 and 5.5 over time as a proxy for pH gradient shape (Figure 4C). The distance between pI markers 4.5 and 5.5 remained stable for IPG-IEF and mixed-bed IEF, while the CA-IEF condition saw a ∼2-fold increase in the distance between pI markers 4.5 and 5.5 during 20 min of IEF (Figure 4C). Additionally, the positions of 5 pI markers between 4.5 and 6.7 remained stable over the course of 30 min IEF using IPG-IEF (Figure 3A). As expected of IPG-based IEF separations, where pH buffering species are covalently fixed in the anticonvective matrix,^26^ pI marker peak positions for both IPG-IEF and mixed-bed IEF remained stable compared to the CA-IEF condition.

**Figure 5:**
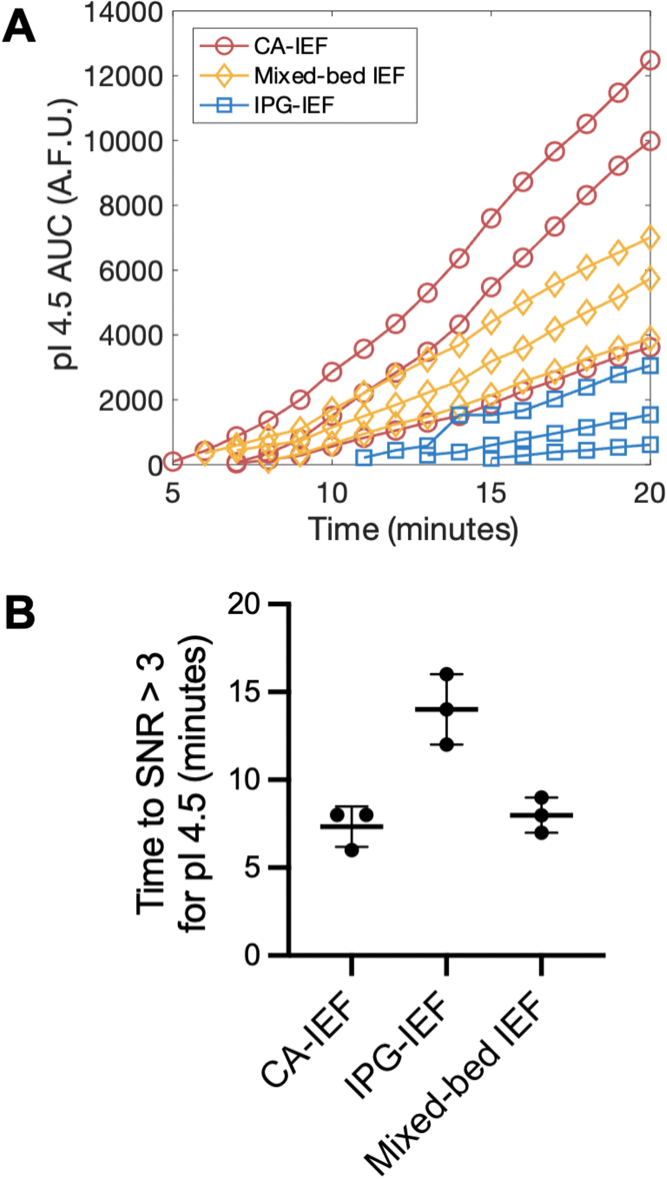
Comparison of the sensitivity and focusing time of IPG-IEF, CA-IEF, and mixed-bed IEF in a microfluidic IEF device. (A) Plot of pI 4.5 marker AUC versus IEF focusing time showing that mixed-bed IEF improves pI marker concentration versus IPG-IEF alone. AUC data from *n* = 3 separations for each IEF condition are plotted for each time point with SNR > 3. (B) Time to SNR > 3 for 3 IEF conditions demonstrating relative focusing speeds.

Lastly, we evaluated the focusing performance of the μIPG device. Using the relative analyte concentration within the focused peaks at several time points during IEF, we can assess the assay sensitivity of the three IEF conditions (i.e., higher analyte concentrations correlate with higher assay sensitivity). The area under the curve (AUC), serving as a proxy for analyte concentration, is measured in arbitrary fluorescence units for pI marker 4.5 in our μIPG device (Figure 5A). At 20 min, the AUC is 8697 ± 4568 for CA-IEF (which we will define as max AUC), 5547 ± 1568 for mixed-bed IEF (64% of max AUC), and 1735 ± 1232 for IPG-IEF (20% of max AUC) (*n* = 3 separations). Additionally, CA-IEF and mixed-bed IEF reached a signal-to-noise ratio (SNR) greater than 3 faster than IPG-IEF (Figure 5B). While IPG-IEF has a more stable pH gradient than CA-IEF (Figure 4), IPG-IEF has poorer focusing performance than CA-IEF with regards to detection sensitivity and focusing time (Figure 5). Mixed-bed IEF has improved focusing performance versus IPG-IEF alone (Figure 5), while retaining pH gradient stability (Figure 4). Differences between the three IEF conditions in AUC at 20 min (Figure 5A) and the time to reach SNR > 3 (Figure 5B) are not statistically significant, as determined using a one-way ANOVA with Kruskal–Wallis test and a posthoc Dunn’s multiple comparison test (*p* < 0.05). However, based on the average results of *n* = 3 separations, our microscale experiments seem to corroborate prior findings that mixed-bed IEF improves detection sensitivity and reduces the focusing time versus IPG-IEF alone.^16^

### Cell Lysate Separations

**Figure 6:**
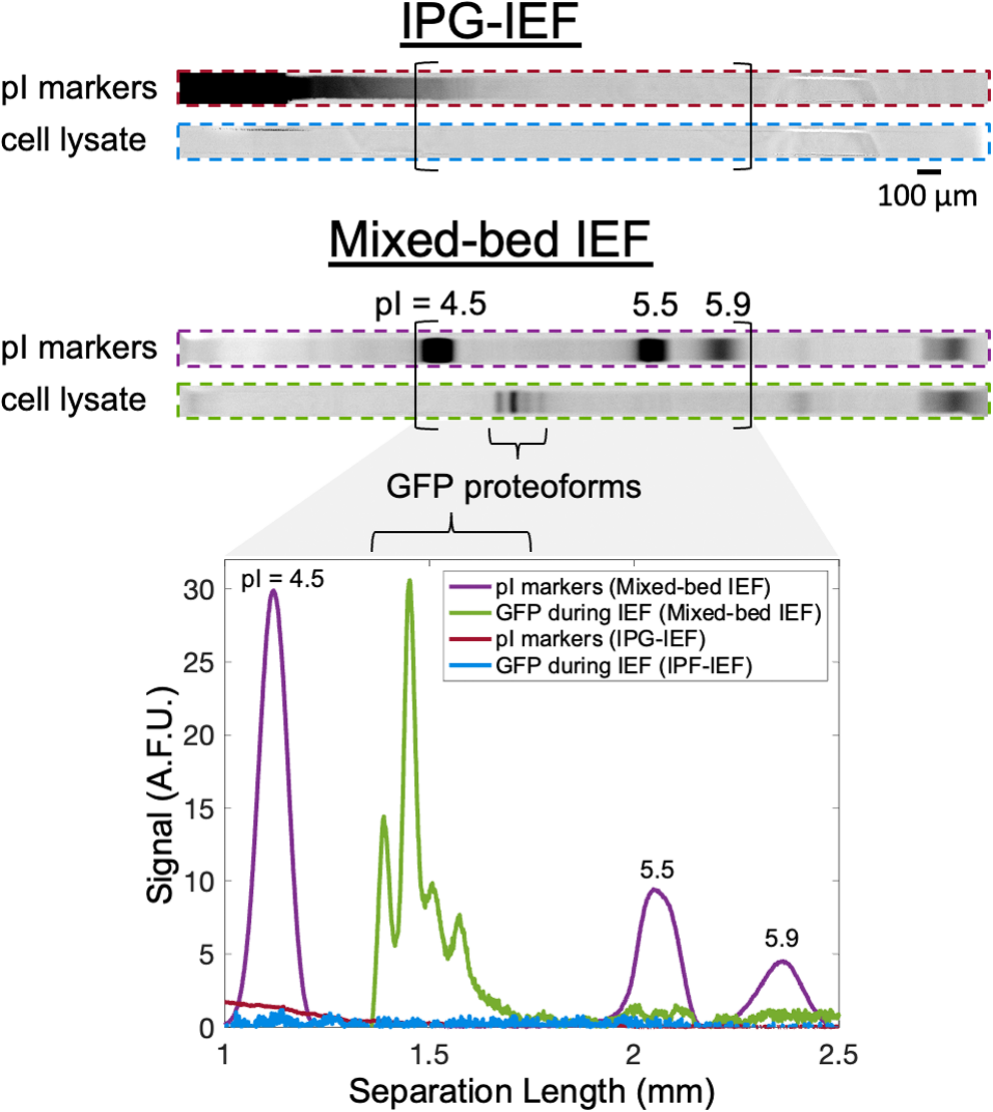
Mixed-bed IEF resolves pI markers and GFP proteoforms from MCF7-GFP cell lysate. Inverted fluorescence micrographs and the corresponding intensity plot of pI markers and GFP focused in the μIPG device from MCF7-GFP cell lysate (40,000 cells). IEF was performed using IPG-IEF or mixed-bed IEF. Profiles were captured after 20 min of IEF. IPG gel is 12%T. Micrographs in the same channel have the same acquisition settings, brightness, and contrast. Representative of *n* = 3 separations for IPG-IEF and *n* = 14 separations for mixed-bed IEF.

To understand the performance of the μIPG device with a complex protein sample, we performed IEF of MCF7-GFP cell lysate. Since the cell lysate did not undergo any purification steps prior to IEF, the cell lysate protein sample would contain all cell proteins solubilized by the lysis buffer. For our purposes, we focused on the analysis of GFP proteoforms since GFP’s fluorescence, arising from a three amino acid chromophore,^42^ provides facile identification of the protein’s location and identity. IEF was performed using both a 6%T IPG gel formulation (Figure 1C) and a 12%T IPG gel formulation (Figure 6). Cell lysate IEF experiments were first performed using IPG-IEF, yet IPG-only IEF did not successfully resolve the pI markers or GFP proteoforms (Figure 6). We hypothesized that the salts in the cell lysate buffer could be interfering with IEF in the IPG-only device,^43^ so we tested mixed-bed IEF in the μIPG device (Figure 6). Mixed-bed IEF is beneficial in samples containing high salt levels where the presence of CA in the sample improves buffering.^43^ Mixed-bed IEF is achieved by the addition of 1% CAs to the IPG gel, which we accomplished by incubating the μIPG device in a sample loading buffer containing 1% CAs overnight.

IEF of MCF7-GFP cell lysate in the μIPG device using mixed-bed CA-IEF resolved up to four GFP proteoforms (Figure 6). Out of 14 independent IEF separations (technical replicates), 1 separation detected only one GFP proteoform, 6 separations resolved two GFP proteoforms, 3 separations resolved three GFP proteoforms, and 4 separations resolved four GFP proteoforms (Figure S3). Variation in the number of proteoforms resolved could be attributable to the manual fabrication of μIPG devices, as discussed in the pH Gradient Stability and Performance of the μIPG Device subsection. Additionally, variation in the distance between electrodes during μIPG operation could also lead to variability in electric field strengths between technical replicates. The large size of the reservoirs (3 mm diameter) meant that the distance between the electrodes could vary from 3.5 to 9.5 mm and therefore the final electric field strength could vary from 284 to 771 V/cm. Since separation resolution is dependent on electric field strength,^17^ changes in electric field strength between different separations could lead to peak broadening that would obscure proteoform peaks. Mechanically fixing the distance between electrodes could be an avenue to better control the electric field strength and therefore separation resolution.

Moreover, based on pI marker data showing that the AUC has not yet plateaued at 20 min (Figure 5A), we anticipate that injection of the ∼40 000 cell sample in the reservoirs is incomplete at our 20 min IEF end point. Future experiments monitoring the total GFP distribution in the reservoirs and microchannel over IEF focusing time could help elucidate the proportion of sample injected over time and the time needed to reach maximum focusing for higher sensitivity. The GFP proteoform data (Figures 6 and S3) serve as proof-of-concept that a complex protein sample can be analyzed in a μIPG device with mixed-bed IEF without introducing additional complexity (CAs were loaded diffusively instead of electrophoretically).

By fitting a line to the pI markers, we can estimate the pIs of the GFP proteoforms from the GFP proteoforms’ peak locations.^39^ Using the separations that resolved four proteoforms (Figure S3), the GFP proteoforms’ pIs, from most acidic to most basic, are 4.80 (CV = 0.09%), 4.86 (CV = 0.13%), 4.92 (CV = 0.07%), and 5.00 (CV = 0.266%) (*n* = 4 separations). The GFP proteoforms detected had pIs between 4.8 and 5.0, comparable to the glass-based μIPG device (GFP pIs ranging from 4.88 to 5.19)^28^ and slab-gel IEF (GFP pIs ranging from 4.7 to 5.1).^42^ The various GFP proteoforms have been attributed to differential C-terminal cleavage by nonspecific proteases.^42^ While the core of the GFP protein is resistant to proteolysis, the C-terminus “tail” sequence, His-Gly-Met-Asp-Glu-Tyr-Lys, contains both basic and acidic amino acid residues, which, when cleaved, produce a variety of GFP proteoforms.^42^ These GFP proteoforms can be detected by various techniques, including isoelectric focusing^44^ and capillary zone electrophoresis.^45,46^

## Conclusions

The design and development of IEF using IPG gels in elastomeric microfluidic devices advances microfluidic analytical tools for targeted proteomic assays by allowing rapid prototyping device design approaches to be adopted for protein separations. The double-photopolymerization procedure reported here for the fabrication of μIPG creates a composite gel that facilitates IEF through the IPG gel component, allowing for resolution of analytes differing by approximately 0.1 isoelectric point. Motivated by the desire to design for enhanced IEF assay throughput, minimizing the separation channel length allows for more separation lanes per unit surface area. The substantial cathodic drift observed in microscale IEF has limited design to these performance goals to date. Recognizing the cathodic drift limitation on microfluidic IEF, we demonstrate that the μIPG device operated using mixed-bed IEF reduces cathodic drift compared to CA-IEF. Further, the mixed-bed IEF configuration allows for analysis of cell lysate not previously reported with microscale IPG-IEF alone. By addressing the limitations of carrier ampholytes and slow-prototype-cycle glass devices, the μIPG device described here offers a promising platform for protein separation and analysis.

Future research can focus on expanding the capabilities of the μIPG by exploring different gel formulations (i.e., to modify the pH gradient). Additionally, efforts can be directed toward enhancing the sensitivity and resolution of the μIPG for the analysis of complex protein samples, enabling the detection and characterization of rare or low-abundance proteoforms. Optimization of the total number of cells added to the device would be needed for analysis of low-abundance proteoforms. Moreover, the flexibility of PDMS allows for the creation of complex microfluidic designs and the integration of various features, such as channels, valves, and chambers, in a single device. We anticipate that the PDMS-based μIPG device presented here could be integrated with other PDMS-based microfluidic devices for additional capabilities (i.e., to automate sample preparation). Lastly, the reversible bond between PDMS and glass could be used to expose the IPG gel for immunostaining of proteins.^31^ Immunostaining is an effective strategy for increasing the multiplexing capability and sensitivity of electrophoretic assays on complex samples for targeted proteomics. Immunostaining allows for the imaging of various species in separate channels, which allows the separation channel to remain short since species in separate channels can overlap in space.^47^

With an ability to analyze complex biological samples and achieve high-resolution protein separations, the μIPG device holds great potential for advancing the field of targeted proteomics and undergirding further miniaturization of analytical techniques.

## Supporting Information Available

Supplemental experimental section; micrographs of PA gel polymerization; intensity plots and micrographs of pI markers 4.5, 5.5, and 5.9 focused in microfluidic IEF devices in CA-IEF, IPG-IEF, and mixed-bed IEF configurations; variability in GFP proteoform profiles; numerical modeling of Nile Blue acrylamide diffusion; recipes of PA gel precursors; incubation buffers for all experiments; and anode inlet and cathode inlet sample components for all isoelectric focusing experiments (PDF)
